# The Insect Type 1 Tyramine Receptors: From Structure to Behavior

**DOI:** 10.3390/insects12040315

**Published:** 2021-04-01

**Authors:** Luca Finetti, Thomas Roeder, Girolamo Calò, Giovanni Bernacchia

**Affiliations:** 1 Department of Life Science and Biotechnologies, University of Ferrara, via Borsari 46, 44121 Ferrara, Italy; fntlcu1@unife.it; 2 Laboratory of Molecular Physiology, Department of Zoology, Kiel University, Christian-Albrechts-Platz 4, 24118 Kiel, Germany; troeder@zoologie.uni-kiel.de; 3 German Center for Lung Research (DZL), Airway Research Center North (ARCN), 24118 Kiel, Germany; 4 Department of Pharmaceutical and Pharmacological Sciences, University of Padova, Via VIII Febbraio, 2, 35122 Padova, Italy; girolamo.calo@unipd.it

**Keywords:** tyramine, octopamine, G protein-coupled receptor, pharmacology, physiology, monoterpenes

## Abstract

**Simple Summary:**

This review aims to describe the type 1 tyramine receptors (TAR1s) in insects with a multidisciplinary approach and might be an important tool for a wide scientific audience, including biochemists, molecular physiologists, ethologists, and neurobiologists with a biological entomology background. In fact, in the last years, TAR1 has received much attention due to its broad general interest. The review is composed of a general introduction about the tyraminergic and octopaminergic systems and the corresponding tyramine (TA) and octopamine (OA) receptors, including the recent classification as well as their brief structural and functional information. The four chapters then describe TAR1s: (1) Molecular and structural characterization, with the purpose to provide a clear biochemical overview of the receptor that ensures a well-defined TAR1 identity; (2) pharmacology, in which a clear TAR1-mediated intracellular signaling pathway is detailed; (3) physiology and behavior, focusing on the TAR1-controlled traits in insects; (4) insecticide target, in which the knowledge on TAR1 roles in insects is associated with the growing evidence about the pest management strategies based on this receptor. The conclusions summarize TAR1 features as well as future directions on which the receptor research should move.

**Abstract:**

Tyramine is a neuroactive compound that acts as neurotransmitter, neuromodulator, and neurohormone in insects. Three G protein-coupled receptors, TAR1-3, are responsible for mediating the intracellular pathway in the complex tyraminergic network. TAR1, the prominent player in this system, was initially classified as an octopamine receptor which can also be activated by tyramine, while it later appeared to be a true tyramine receptor. Even though TAR1 is currently considered as a well-defined tyramine receptor and several insect TAR1s have been characterized, a defined nomenclature is still inconsistent. In the last years, our knowledge on the structural, biochemical, and functional properties of TAR1 has substantially increased. This review summarizes the available information on TAR1 from different insect species in terms of basic structure, its regulation and signal transduction mechanisms, and its distribution and functions in the brain and the periphery. A special focus is given to the TAR1-mediated intracellular signaling pathways as well as to their physiological role in regulating behavioral traits. Therefore, this work aims to correlate, for the first time, the physiological relevance of TAR1 functions with the tyraminergic system in insects. In addition, pharmacological studies have shed light on compounds with insecticidal properties having TAR1 as a target and on the emerging trend in the development of novel strategies for pest control.

## 1. Introduction: The Tyraminergic and Octopaminergic Systems in Insects

In insects, the main biogenic amines are dopamine (DA), serotonin (5-HT), tyramine (TA), and octopamine (OA). Together, they control and modulate a broad range of biological functions essential for the life of the insects. Whereas DA and 5-HT functions and pathways are highly conserved in both vertebrates and invertebrates, TA and OA can be considered the invertebrate counterparts of the catecholamines epinephrine and norepinephrine of vertebrates [1]. TA, OA, and the catecholamines epinephrine and norepinephrine have many features in common: They are synthesized from the same precursor amino acid (tyrosine), share both structural and functional characteristics such as interaction with G protein-coupled receptors (GPCRs), and regulate similar behavioral and physiological traits [2]. To generate OA and TA, tyrosine is decarboxylated by the tyrosine decarboxylase (Tdc), which gives rise to TA. This is then hydroxylated to OA by the tyramine β-hydroxylase (Tβh) [3]. The insect nervous tissues contain high levels of both OA and TA, supporting the view that they act as neurotransmitters [1]. Moreover, they act also as neuromodulators and neurohormones in a wide variety of physiological processes, also operating in a paracrine, endocrine, and autocrine fashion in peripheral organs [4,5]. Originally, TA was considered only as the intermediate product necessary for the synthesis of OA [6]. Nowadays, it is known that TA and OA perform important functions independently of each other [7]. In many cases, TA and OA operate as antagonist modulators in a coordinated way [3] to control important functions in insects, including olfaction [8], locomotion [9], fertilization and reproduction [10], and metabolism [11]. OA and TA exert their physiological actions by interacting with and activating different receptors, the tyramine (TAR) and the octopamine (OAR) receptors [3]. The study of TA and/or OA receptor-deficient animals has revealed that the corresponding receptors play important roles in modulating the biology, physiology, and behavior of invertebrates. In fact, changing the normal function of these receptor classes by blocking or overstimulating them can lead to the death of an insect or interfere with physical fitness and reproductive capacity [12]. These receptors are classified into five main groups based on their primary structure: α1-adrenergic-like receptors (Octα1R, also known as OAMB or OA1), α2-adrenergic-like receptors (Octα2R, also known as OA3), β-adrenergic-like receptors (OctβR, also known as OA2), tyramine type 1 receptors (TA/OA, Tyr1-R or TAR1), and tyramine type 2 and 3 receptors (Tyr2-R or TAR2 and Tyr3-R or TAR3) [13,14]. To date, the gene coding for TAR3 has only been identified in *Drosophila* [15]. Octα-Rs have structural and functional similarities to mammalian α-adrenergic receptors and are classified in Octα1-R and Octα2-R. Octα1-R, characterized for the first time in *Drosophila melanogaster* [16], is expressed mostly in the insect brain [17] and has been identified in other insects, such as *Apis mellifera* [18], *Periplaneta americana* [19], and *Bombyx mori* [20]. All α-adrenergic-like OA receptors play key roles in appetitive olfactory learning [21], reproduction [22], circadian clock, and sleep modulation [23]. The other OA receptors (Octβ-R) share similarities with mammalian β-adrenergic receptors and are able to directly control different functions, including ovulation [24], locomotor activity [25], and feeding [26]. The TAR1 receptor group, showing a limited selectivity for TA and the ability to couple with G_i_ and G_q_ proteins, is described in the next chapters. On the other hand, TAR2 is thought to be a receptor highly specific for TA, and its activation elicits a selective stimulation of Ca^2+^ release [27]. TAR2 seems to be involved in the regulation of renal function due to its high expression in Malpighian tubules, as well as a direct controller of courtship behaviors in fruit flies [28,29,30,31]. In contrast, TAR3 is activated by TA and, to a lesser extent, by OA, and decreases intracellular cAMP but also increases Ca^2+^ levels [13,15]. The five OARs and three TARs have been shown to be differentially expressed in *Drosophila* [32], therewith confirming their multiple and often unique roles in controlling physiology and behavior in insects. Since OARs and TARs play pivotal roles in insect physiology, they are also possible targets for insecticides used in pest control [33].

## 2. TAR1: Molecular and Structural Characterization

TAR1s, like all GPCRs, consist of a single polypeptide chain containing seven hydrophobic transmembrane domains, connected by six hydrophilic loops, along with an extracellular N-terminal and an intracellular C-terminal region [34]. To date, TAR1s have been characterized in 16 insect species (Table 1).

Recently, it has been reported that *P. americana* expresses a second type 1 tyramine receptor, named PeaTAR1B (accession number: LT900530), in addition to the PeaTYR1 or PeaTAR1A (accession number: AM990461) [42,43]. PeaTAR1B appears structurally related to PeaTAR1A, sharing several biochemical features such as N-glycosylation and P sites, as well as amino acids involved in the binding with TA (Table 1). The first TAR1 was described in *D. melanogaster* and called Tyr-dro [35]. The amino acid sequence, composed by 601 residues, is significantly longer compared to other TAR1s (Table 1). In fact, in *D. melanogaster* TAR1, a putative eighth transmembrane domain was found close to the N-terminal region [35]. The same domain was reported also in *Drosophila suzukii* and *Phormia regina* TAR1 [36,37] but it seems to be exclusive to the order Diptera. As suggested by Baxter and Barker [52], this eighth domain might be a cleavable signal sequence or leader peptide, a sequence that plays a key role during the first steps of the GPCRs intracellular transport [53]. However, the exact function of this domain remains to be clarified. Several sequence motifs essential for correct receptor folding, ligand binding, and signal transduction are well conserved within the TAR1 family. Between the fifth and sixth transmembrane domain, there is the long intracellular loop 3 (IL3) composed of about 150 amino acids [34]. Interestingly, in Diptera, the TAR1 IL3 is longer than in other insects. In particular, IL3 is 237, 238, and 246 residues long in *D. melanogaster*, *D. suzukii*, and *P. regina*, respectively [35,36,37]. In the β-adrenergic receptors, the IL3 is involved in intracellular signaling activation [54]. Given the evolutionary proximity between TAR1 and adrenergic receptors, it cannot be excluded that the IL3 region might play the same role. Braza and colleagues have observed that, in the *Sitophilus oryzae* TAR1, the IL3 region is a very flexible element and is stabilized by TA binding, a key event for signaling with the G-protein [51]. However, *A. mellifera* TAR1 has a relatively short IL3, composed of 110 amino acids. Blenau and colleagues linked this unusual aspect to the fact that this receptor couples only with G_i_ and not also with G_q_ [39]. The GPCRs are subjected to a variety of post-translational modifications among which glycosylation, phosphorylation, and palmitoylation are the most prominent [55]. In all the TAR1s characterized, two putative N-glycosylation sites within the N-terminal region have been identified (Table 1). The number of phosphorylation site ranges from 3 (*D. suzukii* TAR1) to 19 (*Rhodnius prolixus* TAR1) [36,45]. These sites are generally phosphorylated by protein kinase C (PKC) or protein kinase A (PKA), modulating the sensitivity of the receptor to coupling with G proteins [56]. Palmitoylation is the addition of a palmitic acid residue (a 16C saturated fatty acid) and occurs on one or more cysteines on the intracellular side of GPCRs, typically in the C-terminal region [57]. In TAR1s, putative palmitoylation sites have not been identified. This could be explained by the extremely short C-terminal region (15–20 aa) which does not contain cysteine residues. This aspect is shared with α2-adrenergic receptors [58]. In fact, palmitoylation is an event that generally influences the choice of signaling through particular G proteins as well as receptor phosphorylation and internalization [57]. A conserved domain, coding for the amino acids DRY, immediately downstream of the third transmembrane domain, was identified in all TAR1s examined. This motif appears important for the stabilization of GPCRs between inactive and activate conformations and is typical of catecholaminergic receptors. The DRY domain and a glutamate residue in the sixth transmembrane domain create an ionic lock that stabilizes the inactive conformation of the receptor [59]. Through site-directed mutagenesis, Ohta and colleagues were the first to identify the amino acid residues involved in TA binding of *B. mori* TAR1 [60]. In particular, in the mutant D134A the TA-mediated cAMP reduction observed in wild-type *B. mori* TAR1 was completely abolished. Furthermore, the double mutant S218A and S222A was also not able to attenuate cAMP levels after stimulation with TA. The authors suggested a binding scheme by which the carboxylic group of D134 residue forms an ion pair with the protonated amine of TA, while the S218 and S222 are involved in H-bond between the hydroxyl groups.

Through molecular docking approaches, Braza and colleagues confirmed that *S. oryzae* TAR1 binds TA by forming H-bonds with D^114^ (in the third transmembrane domain) and with N^427^ (in the sixth transmembrane domain) [51]. Furthermore, this study reveals that other amino acid residues, such as V^83^, C^118^, W^394^ and S^428^, are involved in TA binding suggesting a more complex binding pocket for TA.

## 3. TAR1: Pharmacology

The characterization of a receptor downstream signaling and cascade requires a precise study of its pharmacological profile. The TAR1 pharmacology is quite intriguing, since it was initially characterized as an OA receptor capable of also interacting with TA. Arakawa was the first to pharmacologically characterize a TAR1 by cloning and expressing the *D. melanogaster* TAR1 in Chinese Hamster Ovary (CHO)-K1 cells [61]. In this study, several biogenic amines were tested as putative agonists, including 5-HT, adrenaline, and OA, but not TA. The authors concluded that the receptor was an OA receptor given its high affinity to OA. However, in two separate studies, the same receptor was further investigated [35,62]. When expressed in mammalian cells (Cos-7), it showed a TA-mediated inhibition of adenylate cyclase activity, proving its G_i_ coupling activity. In particular, TA was able to reduce forskolin-stimulated cAMP levels in a dose-dependent manner with a pEC_50_ of 5.62 (Table 2).

**Figure 1:**
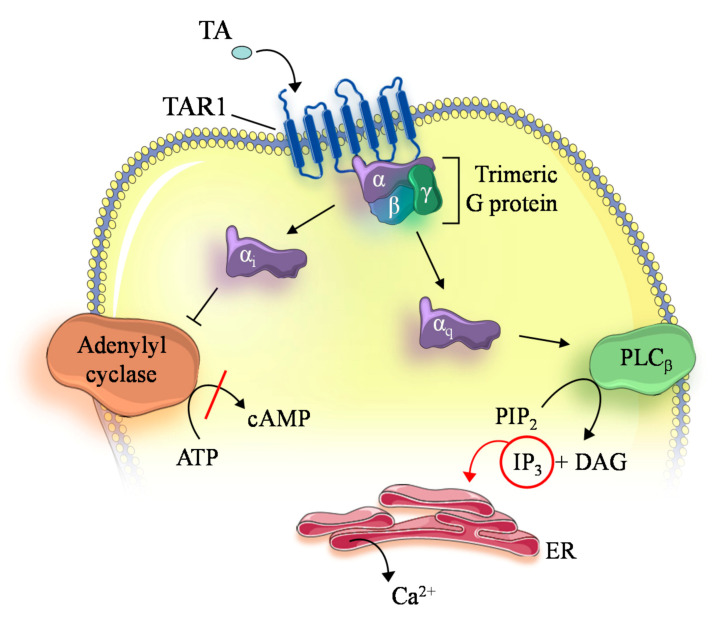
Intracellular signaling pathways trigged by TAR1 activation. ATP (Adenosine triphosphate), cAMP (Cyclic adenosine monophosphate), PLCβ (phospholipase Cβ), PIP2 (Phosphatidylinositol 4,5-bisphosphate), IP3 (Inositol trisphosphate), DAG (Diacylglycerol), ER (Endoplasmatic reticulum).

Conversely, OA was less potent, with a pEC_50_ of 4.52 [35]. Furthermore, Robb and colleagues investigated the TA and OA responses of *D. melanogaster* TAR1 upon cloning into CHO cells. In particular, this work clearly demonstrated that *D. melanogaster* TAR1 is more sensitive to TA than OA and that it activates its signaling cascade not only through G_i_-coupling but also via G_q_ proteins [62]. TAR1 signaling is, therefore, far more complex than initially thought. Over the years, several TAR1s have been cloned and pharmacologically characterized from other insects, providing a well-grounded description of the receptor pharmacology. TA appears to be significantly more potent than OA in activating the receptor in terms of both G_i_ and G_q_-mediated intracellular cascades. In particular, in *A. mellifera*, *D. melanogaster*, and *D. suzukii*, TA appeared to be one order of magnitude more potent than OA, while in *R. prolixus*, *L. migratoria*, *Plutella xylostella*, and *Halyomorpha halys*, TA was twice as effective as OA (Table 2). These variations in potency might be truly species specific or they might be traced back to the different cell lines used: Most of the studies stably expressed TAR1 in the Human Epithelial Kidney (HEK) 293 cell line, while others used insect cell lines (S2 and Sf9), CHO, or Murine Erythroleukaemia cells. Furthermore, another reason for variation in the pharmacological profile might be linked to the different experimental approaches used. In fact, for each TAR1 studied, either the G_i_- or G_q_-mediated intracellular pathway was investigated, with a preference toward G_i_. The reason for investigating the G_i_-mediated transduction pathway is perhaps due to the fact that the α2-adrenergic receptors are coupled exclusively to G_i_ [58]. Studies investigating the G_q_-mediated intracellular pathway have been performed on TAR1 from *D. suzukii*, *L. migratoria*, *R. prolixus*, and *H. halys* [36,38,45,47]. In the study by Blenau and colleagues, *A. mellifera* TAR1, expressed in HEK 293 cells, was tested for its ability to activate intracellular signaling via both G_i_ and G_q_ proteins, and it was shown to promote its downstream cascade exclusively via G_i_ activation. As discussed above, this receptor peculiarity may be due to the shorter IL3, but further investigation might be necessary [39]. *D. suzukii* TAR1 was the first receptor studied with the dynamic mass redistribution (DMR) assay, an assay able to investigate both the G_i_ and G_q_ contribution to intracellular signaling simultaneously. In this study, DMR assays revealed that TA is able to evoke a positive concentration dependent signal in HEK 293 cells stably expressing the *D. suzukii* TAR1 (pEC_50_ of 6.87), while OA can elicit an intracellular Ca^2+^ release only at 10 µM [36]. *P. americana*, as discussed above, is the only insect presenting two distinct TAR1 [42,43]. In fact, the occurrence of two functional TAR1s with different pharmacological properties has not yet been described for any other insect species. In particular, although both PeaTAR1s were activated by TA via G_i_ coupling, PeaTAR1B displayed a different response to antagonist compounds. In fact, several antagonists completely lacked inhibitory potential on the receptor, such as chlorpromazine [43]. In terms of TAR1 antagonist pharmacological profile, yohimbine showed the highest affinity for this receptor class (Table 2). Yohimbine is an α2-adrenergic receptor antagonist and concentrations up to 1 µM were able to antagonise TA in TAR1s (Table 2). When the *R. prolixus* TAR1 was expressed in HEK 293, the rank order of antagonist potency was yohimbine > metoclopramide > phenoxybenzamine > phentolamine > cyproheptamide > gramine > mianserin > chlorpromazine [45]. Similar results, i.e., yohimbine > mianserin > phentolamine > chlorpromazine, were obtained when investigating the *P. xylostella* TAR1 expressed in HEK 293T cells [47]. When biogenic amines different from TA and OA, such as dopamine, adrenaline, noradrenaline, L-DOPA, and histamine, were tested on TAR1s, no significant agonist effects were observed, suggesting that this receptor class is selectively responsive to TA and OA [40,42,44,45,46,47]. The pharmacological profile of TAR1 has been characterized especially for the antagonist, whereas our knowledge of alternative agonists is almost completely lacking. The preferred receptor coupling between G_i_ and G_q_ proteins, whether TA preferably activates the G_i_- or G_q_-mediated transduction pathway, and how much OA, even though less potent, contributes to this, remain to be elucidated. Nevertheless, it is obvious that TAR1s are promiscuous GPCRs which are able to couple with both G_i_ and G_q_ proteins as summarized in Figure 1.

**Figure 2:**
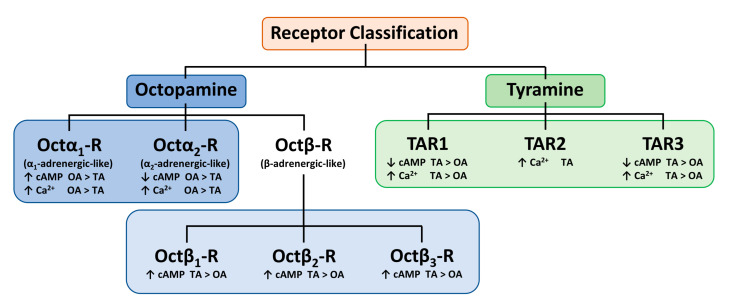
Revised scheme, based on Hana and Lange, describing the tyramine (TA)/octopamine (OA) receptors classification based on their sensitivity to ligands and their downstream effects [45].

Therefore, the TA/OA receptors scheme proposed by Wu and colleagues [13] should be revised, defining TAR1s more sensitive to TA in both Ca^2+^ and cAMP intracellular variations (Figure 2).

## 4. TAR1: Physiology and Behavior

*TAR1* transcript localization analysis provides information on the expression profile of the receptor and helps to better understand its physiological and behavioral functions. In *D. melanogaster*, the receptor is mainly expressed in the central nervous system (CNS) [32]. Through Gal4/UAS technology, *D. melanogaster TAR1* transcripts were found to be abundant in the pars intercerebralis, in the mushroom bodies, and in the antennal and olfactory lobes [66]. A higher expression of *TAR1* in nervous tissues compared to the periphery was also observed in *D. suzukii*, *Chilo suppressalis*, *R. prolixus*, *P. xylostella*, *H. halys*, *Mamestra brassicae*, and *Agrotis ipsilon*, suggesting a crucial role of the receptor in controlling a broad range of physiological functions and behaviors [36,44,45,46,47,48,50]. Interestingly, *TAR1* was also strongly expressed in the antennae of *M. brassicae*, *A. ipsilon*, and *H. halys*, where it could regulate olfactory-mediated behaviors [8,47,48,50]. A possible correlation between TAR1 and olfaction was established for the first time in 2000 by Kutsukake and colleagues [67]. This study characterized a *D. melanogaster* TAR1-defective line, called *honoka*, whose behavioral responses to repellents were reduced in comparison to wild-type flies. Furthermore, using in situ hybridization, Brigaud and colleagues observed that *TAR1* was expressed at the base of the olfactory sensilla trichodea, pheromone-sensitive sensilla, rather than in sensilla chaetica, which are mechano-sensitive in *A. ipsilon* [50]. A similar correlation has been hypothesized in *H. halys*. In the antennae, *TAR1* appeared to be highly expressed in flagellomeres, apical structures rich in sensilla trichoidea which are essential for pheromone perception. Furthermore, RNAi-mediated *TAR1* silencing resulted in a lower sensitivity to the alarm pheromone (*E*) -2-decenal [47], therefore suggesting a pivotal role of this receptor in olfaction-mediated responses. The role of TAR1 in olfactory perception was further confirmed by imaging analysis performed on *A. mellifera*. In two studies conducted in 2017 on the honeybee brain, the authors showed that *TAR1* is mainly expressed at the presynaptic sites of olfactory receptor neurons (ORNs), innervating the antennal lobes and the mushroom bodies, which are essential structures for the olfactory system [68,69]. A similar *TAR1* mRNA localization was observed by Mustard and colleagues via in situ hybridization in honeybees [65]. Furthermore, *TAR1* showed a higher expression in the antennae of pollen foragers in comparison to nurse ones. In contrast, *OAR1* exhibited the opposite expression profile [70]. Therefore, it can be proposed that, in social insects, TAR1 could represent a key element in defining the caste identity and modulating behavioral features such as olfaction [71]. Behavioral alterations caused by TAR1 modulation have been observed in several studies performed with *L. migratoria* and *D. melanogaster*. In locusts, the ratio between *TAR1* and *OAR1* expression levels influenced olfactory preferences during the solitary-gregarious phase transition. In fact, high levels of *TAR1* promoted solitary behavior by inducing the perception of gregarious pheromones as repellent, while RNAi-mediated *TAR1* downregulation in solitary locusts was able to mediate the transition to the gregarious-like behavior [72]. In a subsequent study, the same authors observed that TAR1 mediates the olfactory responses between the solitary-gregarious phases by modulating the *tspo* transport protein [73]. It is evident that TAR1 is not only important in olfactory regulation but also in locomotor control. In *A. mellifera*, movement impairment could be attributed to TAR1 [74], since the topical application of yohimbine on the abdomen caused a massive movement alteration owing to the selective antagonism of the receptor. However, yohimbine also antagonizes TAR2, and further studies are necessary to evaluate which tyramine receptor is essential in this response. A TAR1-mediated role in locomotion has been hypothesized by a few studies reporting a high *TAR1* expression in leg muscles of *D. melanogaster*, *P. americana*, and *A. ipsilon* [32,42,50]. Furthermore, Tβh-deficient flies having no OA but high levels of TA showed a severe locomotion deficit, partially rescued by diet-fed TAR1 antagonist yohimbine [75]. Furthermore, it has been observed that in *Drosophila*, the TA/OA ratio was modulated by nutritional state, such as satiation and starvation. In particular, satiation inhibits locomotion through the increase of TA levels. Moreover, this mechanism controlling locomotor behavior requires a functional TAR1 in *D. melanogaster* [76]. However, these observations do not rule out the possibility that TAR1-mediated movement alterations could be controlled by other nervous areas rich in *TAR1* transcripts such as the central complex [66]. TAR1 has also been shown to influence the gustatory responses. The *D. melanogaster* TAR1^PL00408^ defective line exhibited higher body fat accumulation, starvation resistance, and food intake in comparison to wild-type flies [66,77], thus suggesting that nutritional constraints work through a functional TA-dependent pathway, even if the precise mechanism by which TAR1 modulates these essential metabolic traits is still unknown. Some indications came from *D. melanogaster*, where, like many other insects, lipids are mainly stored in the fat body. Their storage and release are mainly controlled by two hormones, the insulin-like peptides (mainly ILP2) and the adipokinetic hormone (AKH, analogous to the mammalian glucagon) [11]. Under acute stress, mobilization of lipids from the fat body is essential for survival. This mechanism also appears to be controlled by both OA and TA, presumably through the modulation of ILP2 secretion [78,79]. Therefore, the increased triglycerides (TG) level observed in TAR1^PL00408^, as compared to control flies, might be related to a direct tyraminergic action on the ILP2 release. RNAi-mediated *D. melanogaster TAR1* silencing, targeted to the fat body, indeed triggered a ILP2 reduction in insulin-producing cells, located in the pars intercerebralis, and an increased TG accumulation [77], confirming a significant role for TAR1 in lipid metabolism. Furthermore, the increased TG levels in TAR1^PL00408^ flies correlates well with other traits observed, such as enhanced food intake, lower movement propensity, and lower metabolic rate [66]. It has recently been proposed that TAR1 could be involved in processes related to sugar sensibility and food intake regulation. *honoka* flies, a *D. melanogaster* TAR1-defective line, exhibited a higher starvation resistance but, in contrast to TAR1^PL00408^ flies, a reduced responsiveness to sugar stimuli compared with control flies [80]. It is worth noting that *TAR1* is highly expressed in neurons located in the sub-esophageal ganglia that are presumably associated with the salivary glands and neck muscles control, thus linking TAR1 with feeding. In honeybees, the topical administration of TA induced an increased Gustatory Response Score (GRS) that was sensitive to yohimbine [81]. LeDue and colleagues found that the *TAR1* knockdown in *D. melanogaster* significantly reduced bitter sensitivity in starved flies, suggesting that TAR1 might be directly involved in the gustatory behaviors [82]. Furthermore, foraging honeybees showed a higher GRS as well as higher *TAR1* expression level in the fat body in comparison to nurses, suggesting a correlation between the receptor and sugar responsiveness [83]. Last but not least, in both *D. suzukii* and *R. prolixus*, *TAR1* is expressed in the reproductive organs [36,45]. In particular, *R. prolixus* ovaries display higher *TAR1* transcript levels in comparison to other reproductive tissues such as the lateral oviduct and common oviduct, suggesting its importance in modulating reproductive processes [45]. However, the *D. suzukii* male abdomens showed a significant difference in the *TAR1* expression levels as compared to females, suggesting a possible role of TA in male reproductive system [36]. Recently, *TAR1* gene has been successfully downregulated through RNAi approaches in *A. mellifera* and *H. halys* to investigate the receptor physiological functions [47,84,85]. Thus, biotechnological techniques such as RNAi and CRISPR-Cas9, targeting TAR1, might be useful to better dissect out the TAR1 roles in controlling specific behavior in insects.

## 5. TAR1: Insecticides Target

In addition to their role in the physiology and behavioral control of insects, TAR1s have proven to be interesting targets for insecticides. Amitraz is an acaricide and non-systemic insecticide that targets OA receptors [86]. However, recent studies have demonstrated that amitraz can also exert its toxic effect through TAR1 [44,87]. When the *C. suppressalis* TAR1 was expressed in HEK 293 cells, 10 µM of amitraz was able to inhibit forskolin-stimulated intracellular cAMP, mimicking TA effects [44]. Amitraz was initially thought to work only on OA receptors. However, TAR1s have been wrongly classified as OA receptors [37,88]. Through phylogenetic analyses, Baron and colleagues classified the receptor as Oct /Tyr. In Farooqui’s review, TAR1 was described as a TA receptor [89,90]. On the other hand, ambiguities and annotation errors still persist in public databases. Further evidence supporting the hypothesis that amitraz could interact with TAR1 was provided by Gross and colleagues on *Rhipicephalus microplus* TAR1. When expressed in the CHO cell line, TAR1 was allosterically positively modulated by BTS-27271, an amitraz metabolite [91]. Even if it remains to be elucidated whether the biological effects of the insecticide are really due to the activation of TAR1, it has been shown that two amino acid substitutions in the *R. microplus* TAR1 (T8P and L22S) could be responsible for a lower susceptibility, or even resistance, to the amitraz insecticide action [88], supporting the hypothesis that the amitraz-mediated toxicity is mediated by TAR1. The tyraminergic and octopaminergic systems are interesting targets for natural insecticides such as monoterpenes [92]. These molecules are the main components of plant essential oils and have long been used as phagodeterrents and biopesticides in the pest control [93]. In the last few years, several studies have shown that the monoterpenes can directly activate TAR1. Enan [63] was the first to describe an agonist effect of several monoterpenes (thymol, carvacrol, α-terpineol, eugenol) on the *D. melanogaster* TAR1. However, the same monoterpenes exhibited a different pharmacological effect on D. suzukii and *R. microplus* TAR1 receptors. In fact, they appeared able to increase the TA potency acting as positive allosteric modulators and not as agonists [36,94]. This allosteric modulation of TAR1 was shown to interfere with the receptor expression and subsequently with the insect physiology and behavior [61]. Recent data have revealed that in silico prediction of the structural interaction between monoterpenes and *S. oryzae* TAR1 might provide new insights and possibly new molecules for TAR1-related pest control [95].

## 6. Conclusions and Future Perspectives

Tyramine receptor 1 appears central in controlling physiological processes and defining specific behavioral traits in insects. In recent years, the number of molecular and physiological data have helped shed some light on the role of this receptor. However, despite its importance in controlling the different physiological mechanisms of insects, many questions about the interconnection between the tyraminergic and octopaminergic systems in the modulation of physiology and behavior remain. An intriguing hypothesis suggests that also the ratio between TA and OA receptors might influence behavioral decisions and physiological states. This aspect is evident in social insects, including honeybees, in which both the levels of TA and OA and the expression patterns of the TA and OA receptors seem to influence the caste identities, characterized by well-defined physiological and behavioral profiles [65,66,96]. Moreover, the capacity of insects to adapt to particular environmental events or to food deprivation could also be influenced by this complex tyraminergic system and deserves to be further investigated. Finally, little is known on the interactions between the tyraminergic and other hormonal systems, such as the dopaminergic and insulin, and on the corresponding elicited responses in insects. For this reason, exploring biogenic amine receptors appears essential to understand the complex nervous flexibility typical of many insects. Much remains to be understood on the role of TAR1 in insects and its value as a potential target for biopesticides.

## Figures and Tables

**Table 1:** Molecular features of TAR1s cloned from insects.

Species	Accession number	Amino Acid Sequence Lenght	Residues Interacting with TA	N-Linked Glycosylation	P Sites	IL3 Lenght	Reference
*D. melanogaster*	AAA28731	601	/	N^11^; N^57^	T^136^; T^296^; S^375^; S^397^; S^406^; S^482^; S^507^	237	[35]
*D. suzukii*	MK405664	600	D^187^; S^271^; S^272^; S^275^	N^11^; N^55^	S^420^; S^506^; S^519^	238	[36]
*P. regina*	AB621975	607	/	/	/	246	[37]
*L. migratoria*	X69520	484	D^130^	N^13^; N^198^	T^78^; T^164^; T^238^; T^300^; S^304^; S^365^; S^372^	174	[38]
*A. mellifera*	AJ245824	399	D^116^; S^200^: S^201^; S^204^	N^2^	T^63^; T^149^; T^223^; S^241^; T^265^; S^291^; S^292^; T^296^	110	[39]
*B. mori*	X95607	479	D^134^; S^218^; S^222^	N^11^; N^16^	T^81^; T^241^; T^258^; T^302^	162	[40,41]
*H. virescens*	CAA64864	477	D^132^	N^11^; N^16^	T^78^; T^238^; T^298^; T^302^	165	[40]
*P. americana*	AM990461	441	D^115^; S1^99^; W^381^; N^418^	N^12^,N^17^	T^61^; T^222^; S^275^; S^285^; S^326^; T^334^; S^341^	144	[42]
*P. americana*	LT900530	481	D^120^; S^204^; S^208^	N^7^; N^17^	S^64^; T^153^; T^227^; S^238^; S^252^; T^279^; T^280^; S^282^; T^289^; S^300^; T^350^; T^351^; S^354^; S^379^; S^398^; P^408^	188	[43]
*C. suppressalis*	AFG26689.1	478	D^135^; S^219^; S^223^	N^11^; N^16^; N^347^	T^205^; T^267^; S^274^; T^304^; S^315^; T^371^; S^396^	170	[44]
*R. prolixus*	MF377527	447	/	N^14^; N^17^	T^75^; T^235^; S^246^; S^265^; S^271^; S^274^; S^295^; S^298^; S^311^; S^319^; S^320^; S^322^; S^338^; T^354^; S^371^; S^373^	161	[45]
*P. xylostella*	MK166023	467	D^127^; S^211^; S^215^	N^5^; N^10^	S^252^; S^268^; S^271^; T^296^; S^307^; S^322^; S^349^; S^352^; S^385^	168	[46]
*H. halys*	MT513133	449	D^128^; S^212^; S^213^; S^216^	N^11^; N^14^; N^22^	S^24^; T^30^; T^161^; T^235^; S^246^; S^260^; S^294^; S^319^; S^321^; S^364^	147	[47]
*M. brassicae*	AF343878	477	D^136^	/	/	174	[48]
*P.xuthus*	AB182633	475	D^131^	/	/	171	[49]
*A. ipsilon*	FJ640850	477	D^149^; S^216^; S^217^; S^220^	N^11^; N^16^; N^345^	T^79^; T^165^; T^239^; T^265^; S^314^; S^333^; S^383^	177	[50]
*S. oryzae*	A0A0S1VX60	455	V^83^; D^114^; C^118^; W^394^; N^427^; S^428^	/	/	158	[51]

**Table 2:** Functional and pharmacological properties of TAR1s cloned from insects.

Species	G-Protein	pEC_50_ TA	pEC_50_ OA	Cell Line Used	Antagonist	Reference
*D. melanogaster*	Gi	5.62	4.52	Cos-7	Yohimbine (tested at 1 µM)	[35]
	Gi	5.24	/	S2	/	[63]
*D. suzukii*	Gq	6.35	Detectable to 10 µM	HEK 293	Yohimbine: pA_2_ 7.87	[36]
	Gq+Gi	6.86			Yohimbine: pA_2_ 7.24	
*L. migratoria*	Gq	7.33	Detectable to 10 µM	Murine Erythroleukaemia	Yohimbine (tested at 2.5 µM)	[64]
	Gi	8.40	/		/	
*A. mellifera*	Gi	6.86	5.56	HEK 293	/	[39]
	Gi	7.07	/	Sf9	/	[65]
*B. mori*	Gi	8.28	5.85	HEK 293	Yohimbine > Chlorpromazine > Metoclopramide > Mianserin (tested at 10 µM)	[40]
*P. americana*	Gi	6.46	/	HEK 293	Yohimbine and Chlorpromazine > Mianserin (tested at 10 µM)	[42]
*P. americana*	Gi	8.20	/	HEK 293- CNG	Yohimbine: pA_2_ 6.13 Mianserin: pA_2_ 6.06	[43]
*C. suppressalis*	Gi	6.43	6.01	HEK 293	Yohimbine > Chlorpromazine > Cyproheptadine (tested at 10 µM)	[44]
*R. prolixus*	Gq	7.29	5.16	HEK 293- CNG	Yohimbine > Metoclopramide > Phentolamine > Cyproheptamide > Gramine > Mianserin > Chlorpromazine (tested at 10 µM)	[45]
*P. xylostella*	Gi	6.35	4.86	HEK 293T	Yohimbine > Mianserin > Phentolamine > Chlorpromazine (tested at 10 µM)	[46]
*H. halys*	Gq	5.99	4.41	HEK 293	Yohimbine: pA_2_ 8.26	[47]
